# Clinical effects of traditional Chinese herbal medicine management in patients with COVID-19 sequelae: A hospital-based retrospective cohort study in Taiwan

**DOI:** 10.7150/ijms.96575

**Published:** 2024-05-13

**Authors:** Po-Chun Hsieh, Chih-Chin Yu, I-Shiang Tzeng, Tsung-Han Hsieh, Chiu-Feng Wu, Li-Fan Ko, Chou-Chin Lan, You-Chen Chao

**Affiliations:** 1 Department of Chinese Medicine, Taipei Tzu Chi Hospital, Buddhist Tzu Chi Medical Foundation, New Taipei City, Taiwan.; 2 School of Post-Baccalaureate Chinese Medicine, Tzu Chi University, Hualien, Taiwan.; 3 Department of Research, Taipei Tzu Chi Hospital, Buddhist Tzu Chi Medical Foundation, New Taipei City, Taiwan.; 4 Department of Nursing, Taipei Tzu Chi Hospital, Buddhist Tzu Chi Medical Foundation, New Taipei City, Taiwan.; 5 Division of Pulmonary Medicine, Taipei Tzu Chi Hospital, Buddhist Tzu Chi Medical Foundation, New Taipei City, Taiwan.; 6 School of Medicine, Tzu-Chi University, Hualien, Taiwan.; 7 Department of Internal Medicine, Taipei Tzu Chi Hospital, Buddhist Tzu Chi Medical Foundation, New Taipei City, Taiwan.

**Keywords:** COVID-19 sequelae, traditional Chinese herbal medicine, CAT, CFQ-11, BSRS-5

## Abstract

**Introduction:** An estimated 43% of COVID-19 patients showed sequelae, including fatigue, neurocognitive impairment, respiratory symptoms, and smell or taste disorders. These sequelae significantly affect an individual's health, work capacity, healthcare systems, and socioeconomic aspects. Traditional Chinese herbal medicine (TCHM) management showed clinical benefits in treating patients with COVID-19 sequelae. This study aimed to analyze the effects of personalized TCHM management in patients with COVID-19 sequelae.

**Methods:** After the COVID-19 outbreak in Taiwan, we recorded Chronic Obstructive Pulmonary Disease Assessment Tool (CAT), Chalder Fatigue Questionnaire (CFQ-11), and Brief Symptom Rating Scale (BSRS-5) to assess post-COVID respiratory, fatigue, and emotional distress symptoms, respectively. In this study, we retrospectively reviewed the medical records between July 2022 and March 2023. We analyzed the effects of TCHM administration after 14- and 28-days of treatment.

**Results:** 47 patients were included in this study. The results demonstrated that personalized TCHM treatment significantly improved the CAT, CFQ-11, and BSRS-5 scores after 14 and 28 days. TCHM alleviated physical and psychological fatigue. In logistic regression analysis, there was no statistically significant differences in the severity of the baseline symptoms and TCHM administration effects concerning the duration since the initial confirmation of COVID-19, sex, age, or dietary preference (non-vegetarian or vegetarian).

**Conclusions:** Our study suggested that personalized TCHM treatment notably reduced fatigue, respiratory and emotional distress symptoms after 14- and 28-days of treatment in patients with COVID-19 sequelae. We propose that TCHM should be considered as an effective intervention for patients with COVID-19 sequelae.

## Introduction

Since the beginning of the coronavirus disease 2019 (COVID-19) pandemic in December 2019, more than 767 million patients have been infected with severe acute respiratory syndrome coronavirus 2 (SARS-CoV-2) 1. Although COVID-19 primarily presents as a respiratory infection, it is also acknowledged as a systemic disease affecting organs beyond the lungs. These organs include the cardiovascular system, central and peripheral nervous system, musculoskeletal system, immune system, and other systems, making it a multi-organ systemic disorder 2. A previous meta-analysis reported that the estimated global prevalence of COVID-19 sequelae was 43% (37% among male patients and 49% among female patients) 3. After 6 to 12 months following acute infection with SARS-CoV-2, the COVID-19 sequelae with the highest prevalence were fatigue (37.2%), neurocognitive impairment (31.3%), respiratory symptoms (30.2%), and smell or taste disorder (23.6%) 4. These disorders significantly impact individual health condition, working capacity, the healthcare system, and socioeconomic aspects 4, 5. Current diagnostic and treatment options for COVID-19 sequelae are insufficient, while supportive care is considered the primary approach. Therefore, developing effective and safe management strategies for COVID-19 sequelae is crucial.

Traditional Chinese herbal medicine (TCHM), such as Jing Si Herbal Tea (JSHT) 6, NRICM101 7, NRICM102 8, and Jing Guan Fang 9, exhibits clinical benefits in combating infection with SARS-CoV-2 10. As for COVID-19 sequelae, An *et al.* observed lower white blood cell count (WBC), serum interleukin-6, and procalcitonin levels, as well as higher levels of red blood cells, hemoglobin, platelet count, prealbumin, and albumin in patients undergoing TCHM treatment, compared to those not undergoing TCHM treatment 11. Zhong *et al.* found that a personalized TCHM treatment regimen for COVID-19 rehabilitation improved the clinical symptoms and lung functions and achieved body constitution balance 12. These studies demonstrated the treatment potential of TCHM administration on COVID-19 sequelae.

This hospital-based retrospective cohort study aimed to evaluate the clinical effects of personalized TCHM treatment in patients with COVID-19 sequelae in a real-world setting in Taiwan.

## Materials and Methods

### Ethics approval and consent for participation

The study protocols and procedures were approved by the Institutional Review Board of Taipei Tzu Chi Hospital (Buddhist Tzu Chi Medical Foundation, New Taipei City, Taiwan; Protocol No. 12-XD-038). The requirement for informed consent was waived because it utilized secondary de-identified information.

### Clinical parameters

Due to the COVID-19 outbreak in Taiwan, we used the following scales since July 2022 in the Traditional Chinese Medicine (TCM) outpatient department (OPD) at Taipei Tzu Chi Hospital to objectively assess the COVID-19 sequelae: the Chronic Obstructive Pulmonary Disease (COPD) Assessment Tool (CAT) for respiratory symptoms 13, 14, Chalder Fatigue Questionnaire (CFQ-11) for fatigue symptoms 15, 16, and Brief Symptom Rating Scale (BSRS-5) for emotional distress symptoms 17, 18. These scales have been applied to previous post-COVID symptoms research 14, 15, 17. We recorded the scores in the electronic medical records.

### Study design and cohort selection criteria

This hospital-based retrospective cohort study aimed to evaluate the follow-up clinical effects of individualized TCHM treatment regimen in outpatients with COVID-19 sequelae after 14 and 28 days of treatment. We focused on the fatigue, respiratory, and emotional distress symptoms, which are reportedly the most prevalent COVID-19 sequelae 4. The TCHM regimens (comprising formulas and single herbs) were prescribed under the Taiwan's National Health Insurance system 19 and provided as commercial concentrated extract powder, which were purchased from Taiwan's GMP Pharmaceutical Companies (including Ko Da [Taoyuan]^20^, Kaiser [Tainan]^21^, Sun Ten [New Taipei]^22^, and Sheng Chang [Taipei]) 23.

We first screened the Electronic Medical Records Database to identify outpatients diagnosed with COVID-19 sequelae (International Classification of Diseases, Tenth Revision [1CD-10]: U09.9) who visited the TCM OPD at Taipei Tzu Chi Hospital between July 1, 2022 and March 31, 2023. Second, we excluded the patients who visited the TCM OPD less than three times and those aged less than 18 years. Next, we performed a thorough assessment of electronic medical records to identify patients with recorded biochemical and hematological indicators, along with CAT 14, CFQ-11 15, and BSRS-5 17 values during their initial visit to the TCM OPD (D0) and subsequent follow-up visits 14 and 28 days after Day 0 (D14 and D28, respectively). Those fulfilling the inclusion criteria were included for additional data collection.

### Data collection

We collected the following information from the included patients: (1) demographic characteristics (age, sex, COVID-19 diagnosis date [Dx], the date of the initial visit to the TCM OPD [D0], and dietary habits [vegetarian or non-vegetarian]); (2) values of the biochemical and hematological parameters (as listed in Table 1) on D0; (3) values of the clinical parameters of the questionnaires (CAT, FCQ-11, and BSRS-5) on D0, D14, and D28; (4) the TCHM formulas and single herbs used for the management of COVID-19 sequelae.

### Statistical analysis software

Statistical analyses were conducted using GraphPad Prism 10 for macOS (Version 10.0.1 (170), GraphPad Software, San Diego, CA, USA, www.graphpad.com). Baseline demographic and clinical characteristics are presented as number of patients (%) and medians (interquartile range, IQR). R Statistical Software (Version 4.2.2) was used to conduct McNemar's test. All P values less than 0.05 to indicate statistical significance.

### Intragroup comparisons

We divided the patients into low- and medium-to-high-grade symptom groups based on different clinical parameters (CAT, CFQ-11, and BSRS-5). For CAT, scores ≥ 10 indicating a moderate to very high respiratory symptoms, were used as a cut-off.^14^ For CFQ-11, scores ≥ 16 indicating increased fatigue, were used as a cut-off.^24^ For BSRS-5, scores ≥ 6 indicating emotional distress were used as a cut-off.^17^ We conducted intragroup comparisons of the CAT, CFQ-11, and BSRS-5 values on D0, D14, and D28 using the Friedman test. We also conducted intragroup comparisons of the values of the CFQ-11 items 1-7 (representing physical fatigue) and CFQ-11 items 8-11 (representing psychological fatigue) on D0, D14, and D28 using the Friedman test.

### Proportion analysis

Patients were divided into two groups: low-grade and medium-to-high-grade symptom groups, determined by specific threshold values obtained from the CAT (cut-off: 10), CFQ-11 (cut-off: 16), and BSRS-5 (cut-off: 6) assessments. We counted the number of patients in each group and conducted a proportion analysis to investigate the shifts in the proportion on D0, D14, and D28.

### McNemar's test

McNemar's test was utilized to evaluate the statistical significance of the proportion differences between the low- and medium-to-high-grade symptom groups on D0, D14, and D28, respectively.

### Logistic regression

Additionally, logistic regression was performed to examine the association between the categorical dependent variable and the following independent variables of interest: (1) Duration of Dx to D0 (> 90 days vs. ≤ 90 days); (2) sex (female vs. male); (3) age (≥ 60 years vs. < 60 years); and (4) dietary preference (non-vegetarian vs. vegetarian), concerning the baseline severity of the symptoms and the effects of TCHM administration (CAT, CFQ-11, and BSRS-5 changes of D0 to D28).

## Results

### Cohort characteristics

**Figure 1:**
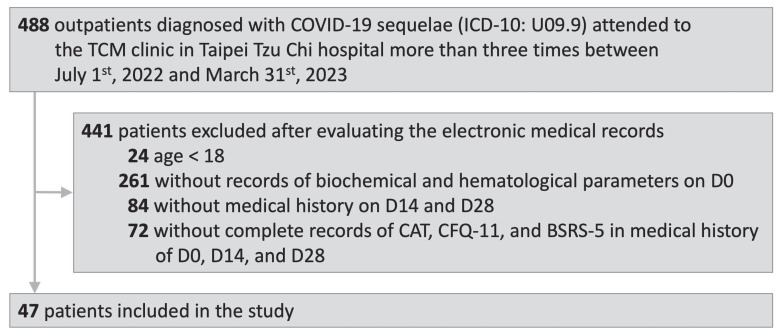
** Study flow diagram.** COVID-19, coronavirus disease-2019; ICD-10, International Classification of Diseases, Tenth Revision; TCM, Traditional Chinese Medicine.

A flow diagram of the study is shown in Fig. 1. After the selection process, 47 outpatients were included in this study. The patients were diagnosed with COVID-19 sequelae (ICD-10: U09.9) and attended to the TCM OPD at Taipei Tzu Chi Hospital between July 1, 2022 and March 31, 2023. The electronic medical records of the included patients documented the biochemical and hematological parameters on D0 and CAT, CFQ-11, and BSRS-5 values on D0, D14, and D28.

The demographic and clinical characteristics of the patients are shown in Table 1. The median age of the patients was 62 years (IQR: 49-70) with a range of 27-83 years. The median duration from Dx to D0 was 37 days (IQR: 19-73) with a range of 8-231 days. Among the 47 patients, 32 (68.1%) were women, 25 (53.2%) were older than 60 years old, and 36 (76.6%) were non-vegetarians. Regarding the baseline clinical parameters, the median CAT, CFQ-11, and BSRS-5 scores were 14 (IQR:11-18), 17 (IQR:14-22), and 5 (IQR:3-10), respectively. Details of the biochemical and hematological parameters are presented in Table 1. All the biochemical and hematological parameters were within the normal ranges.

### Effects of TCHM administration on the respiratory symptoms assessed by CAT

**Figure 2:**
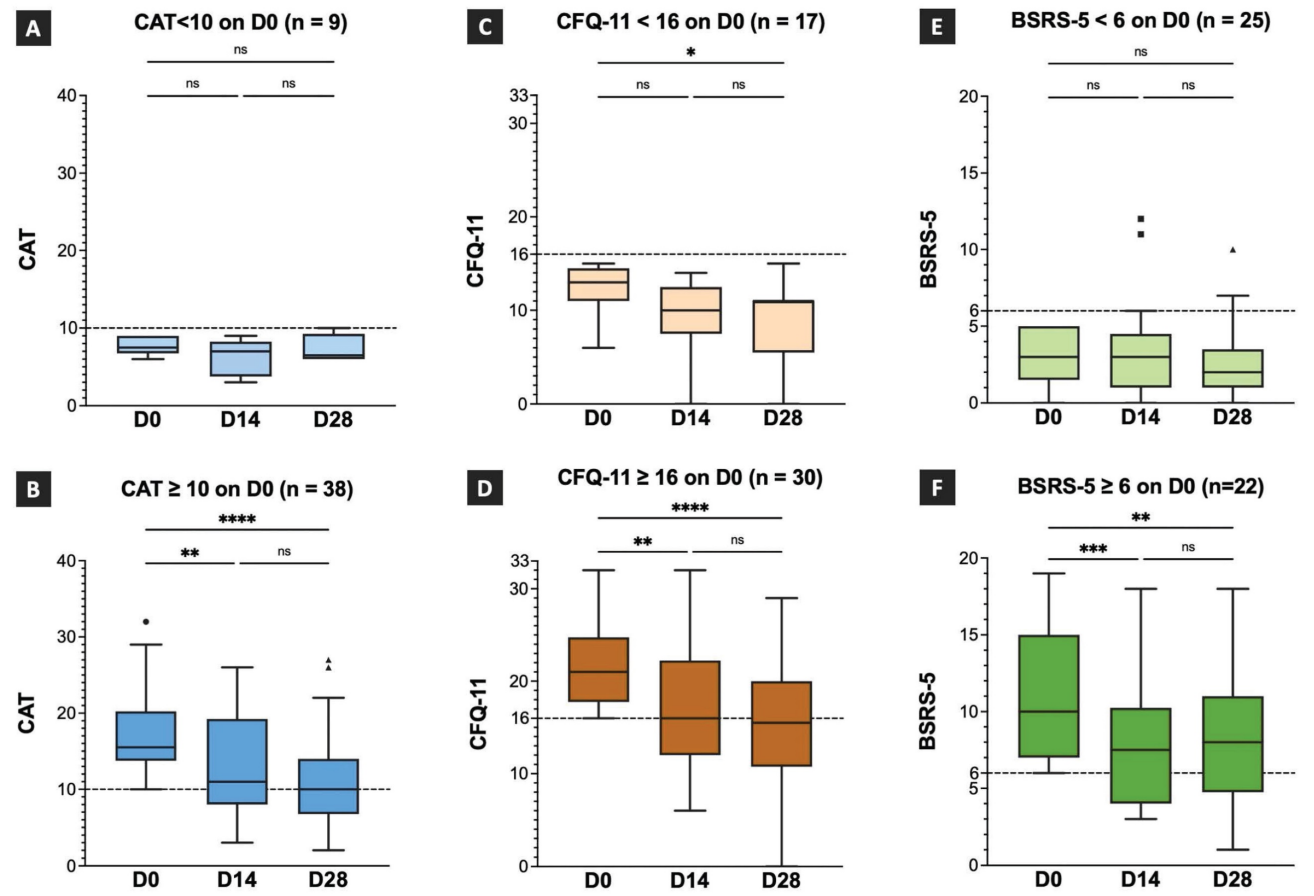
** Effects of TCHM administration on post-COVID respiratory, fatigue, and emotional distress symptoms.** TCHM, Traditional Chinese herbal medicine; COVID-19, coronavirus disease-2019; CAT, Chronic obstructive pulmonary disease Assessment Tool; CFQ-11, Chalder Fatigue Questionnaire; BSRS, Brief Symptom Rating Scale; D0, day of the initial visit to the TCM OPD; D14, day of follow-up visits to the TCM OPD 14 days after D0; D28, day of follow-up visits to the TCM OPD 28 days after D0. * P value < 0.05, ** P value < 0.01, *** P value < 0.001, **** P value < 0.0001

The CAT values on D0, D14, and D28 and the intragroup comparison results are shown in Figure 2A and 2B. In patients with CAT scores of < 10 on D0 (n = 9; Figure 2A), the median CAT score was 7.5 (IQR:6.75-9), 7 (IQR:3.75-8.25), and 6.5 (IQR:6-9.25) on D0, D14, and D28, respectively. No statistically significant difference was observed between the CAT scores on D0 and D14, and D0 and D28 (Figure 2A). In patients with CAT scores more than 10 on D0 (n = 38, Figure 2B), the median CAT score was 15.50 (IQR:13.75-20.23), 11 (IQR:8-19.25), and 10 (IQR:6.7--14) on D0, D14, and D28, respectively. A statistically significant difference was observed between the CAT scores on D0 and D14, and D0 and D28. However, no statistically significant difference was observed between the CAT scores on D14 and D28 (Figure 2B). The results demonstrated that TCHM administration effectively improved the respiratory symptoms, showing significant attenuation after 14 and 28 days of treatment. Despite the absence of a significant difference between the CAT scores on D14 and D28, a notable decrease was observed in the median CAT scores on D28, suggesting a substantial improvement approaching the cutoff threshold.

### Effects of TCHM administration on the fatigue symptoms assessed by CFQ-11

The CFQ-11 scores on D0, D14, and D28 and the intragroup comparison results are showed in Figure 2C and 2D. In patients with CFQ-11 score < 16 on D0 (n = 17, Figure 2C), the median CFQ-11 score was 13 (IQR:11-14.5), 10 (IQR:7.5-12.5), and 11 (IQR:5.5-11) on D0, D14, and D28, respectively. A statistically significant difference was observed between the CFQ-11 scores on D0 and D28. However, no statistically significant difference was observed between the CFQ-11 scores on D0 and D14, and D14 and D28 (Figure 2C). In patients with CFQ-11 scores of > 16 on D0 (n = 30; Figure 2D), the median CFQ-11 score was 21 (IQR:17.75-24.75), 16 (IQR:12-22.25), and 15.5 (IQR:10.75-20) on D0, D14, and D28, respectively. A statistically significant difference was observed between the CFQ-11 scores on D0 and D14, and D0 and D28. However, no statistically significant difference was observed between the CFQ-11 scores on D14 and D28 (Figure 2D). The results demonstrated that TCHM administration effectively improved fatigue symptoms, with significant attenuation after 14 and 28 days of treatment.

Despite the lack of statistically significant differences between the CFQ-11 scores on D14 and D28, the median CFQ-11 score decreased notably to 15.5 on day 28. This marked decrease indicates a substantial improvement in the fatigue symptoms, which was lower than the cutoff threshold that denotes clinically significant amelioration.

**Figure 3:**
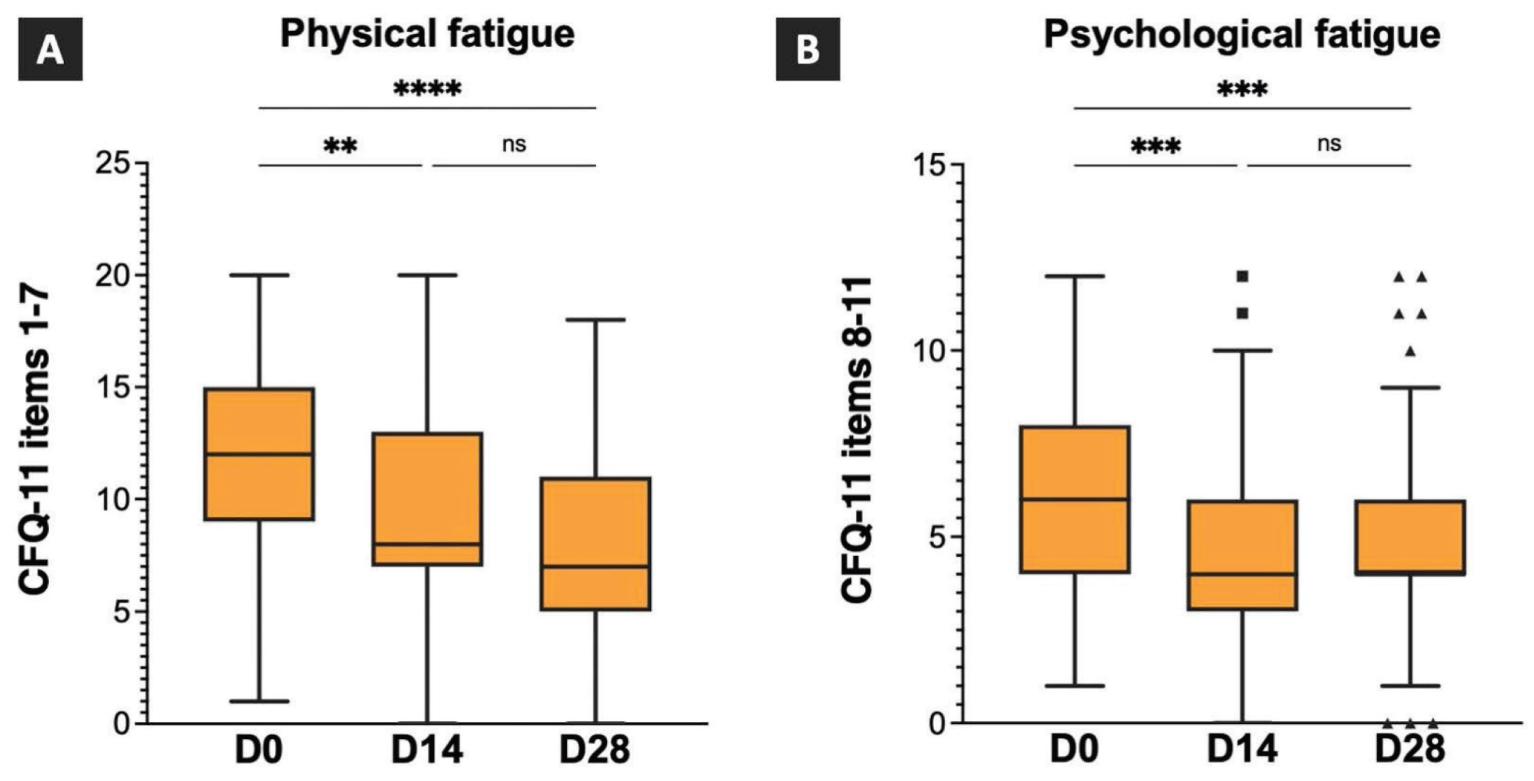
** Effects of TCHM administration on post-COVID fatigue symptoms.** TCHM, Traditional Chinese herbal medicine; COVID-19, coronavirus disease-2019; CFQ-11, Chalder Fatigue Questionnaire, D0: day of the initial visit to the TCM OPD; D14, day of follow-up visits to the TCM OPD 14 days after D0, D28, day of follow-up visits to the TCM OPD 28 days after D0. ** P value < 0.01, **** P value < 0.0001

The scores for the CFQ-11 items 1-7 (physical fatigue) and CFQ-11 items 8-11 (psychological fatigue) on D0, D14, and D28, and the intragroup comparison results are shown in Figure 3. The median physical fatigue scores were 12 (IQR:9-15), 8 (IQR:7-13), and 7 (IQR:5-11) on D0, D14, and D28. A statistically significant difference was observed between the physical fatigue scores on D0 and D14, and D0 and D28. However, no statistically significant differences were observed between the physical fatigue scores on D14 and D28 (Figure 3A). The median psychological fatigue scores were 6 (IQR:4-8), 4 (IQR:3-6), and 4 (IQR:4-6) on D0, D14, and D28, respectively. A statistically significant difference was observed between the psychological fatigue scores on D0 and D14 and D0 and D28. However, no statistically significant difference was observed between psychological fatigue scores on D14 and D28 (Figure 3B). The results revealed significant improvements in both the physical and psychological fatigue symptoms after 14 and 28 days of TCHM administration.

### Effects of TCHM administration on the emotional distress symptoms assessed by BSRS-5

The BSRS-5 scores on D0, D14, and D28 and the intragroup comparison results are showed in Figure 2E and 2F. In patients with BSRS-5 scores < 6 on D0 (n = 25, Figure 2E), the median BSRS-5 score was 3 (IQR:1.5-5), 3 (IQR:1-4.5), and 2 (IQR:1-3.5) on D0, D14, and D28, respectively. No statistically significant differences were observed between D0 and D14, D0 and D28, and D14 and D28. In patients with a BSRS-5 score > 6 on D0 (n = 22, Figure 2F), the median BSRS-5 score was 10 (IQR:7-15), 7.5 (IQR:4-10.25), and 8 (IQR:4.75-11) on D0, D14, and D28, respectively. A statistically significant difference was observed between D0 and D14 and D0 and D28. However, no statistical significance was observed between D14 and D28. The results demonstrated that TCHM administration effectively relieved emotional distress symptoms, showing significant attenuation after 14 and 28 days of treatment. Although no statistically significant difference was observed between the BSRS-5 values on D14 and D28, the median BSRS-5 score notably decreased to 8 on day 28. This marked reduction implies a substantial improvement in the emotional distress symptoms approaching a threshold indicating clinically significant amelioration.

### Effects of TCHM administration on the changes in the proportion of COVID-19 sequelae

**Figure 4:**
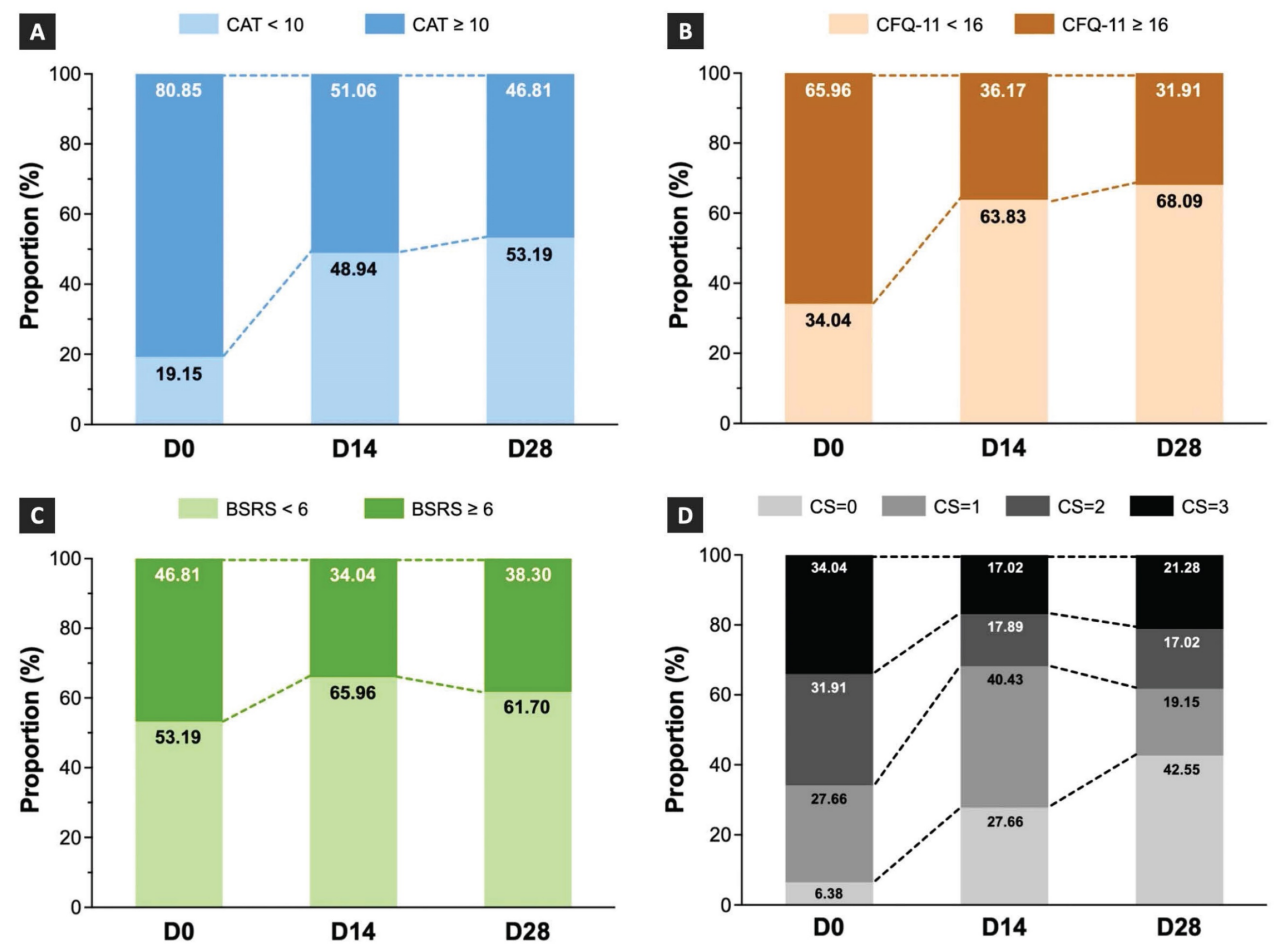
** Shift of the proportions following TCHM administration.** TCHM, Traditional Chinese herbal medicine; CAT, Chronic obstructive pulmonary disease Assessment Tool; CFQ-11, Chalder Fatigue Questionnaire; BSRS, Brief Symptom Rating Scale; D0, day of the initial visit to the TCM OPD; D14, day of follow-up visits to the TCM OPD 14 days after D0; D28, day of follow-up visits to the TCM OPD 28 days after D0.

The proportions of patients assigned to the low- and medium-to-high-grade symptom groups on D0, D14, and D28 are depicted in Figure 4. The results showed that the proportion of patients in the medium-to-high-grade symptom group assessed using CAT was 80.85% on D0, which decreased to 51.06% and 46.81% on D14 and D28, respectively (Figure 4A). The results indicated that the proportion of patients in the medium-to-high-grade symptom group assessed by the CFQ-11 was 65.96% on D0, which decreased to 36.17% and 31.91% on D14 and D28, respectively (Figure 4B). The results showed that the proportion of patients in the medium-to-high-grade symptom group assessed using the BSRS-5 was 46.81% on D0, which decreased to 34.04% on D14 and slightly increased to 38.30% on D28 (Figure 4C).

As patients with COVID-19 sequelae often present co-occurrence symptoms from different categories, we also calculated the proportion of combined symptoms involving respiratory, fatigue, and emotional distress conditions. The results demonstrated that the proportion of patients with three types of co-occurrence symptoms was 34.04% on D0, which decreased to 17.02% on D14 and slightly increased to 21.28% on D28 (Figure 4D). The proportion of patients with two types of co-occurrence symptoms was 31.91% on D0, which decreased to 17.89% and 17.02% on D14 and D28, respectively (Figure 4D). The proportion of patients with one type of symptom was 27.66% on D0, which increased to 40.43% on D14 and decreased to 19.15% on D28 (Figure 4D).

We conducted the McNemar's test to assess the statistical significance of the proportional differences between the low- and medium-to-high-grade symptom groups on D0, D14, and D28. The findings demonstrated significant differences in the respiratory and fatigue symptoms between the D0 and D14 and D0 and D28 time points (Table 2). The results revealed that TCHM significantly decreased the proportion of patients with respiratory and fatigue symptoms after 14 and 28 days of treatment.

### Differences in the severity of the baseline symptoms and TCHM administration effects based on different predictors

We conducted a logistic regression analysis to assess the differences in the baseline severity of the symptoms and the effects of TCHM administration (CAT, CFQ-11, and BSRS-5 changes from D0 to D28) using the following predictors: (1) Duration of Dx to D0 (> 90 days vs. ≤ 90 days); (2) sex (female vs. male); (3) age (≥ 60 years vs. < 60 years); and (4) dietary preference (nonvegetarian vs. vegetarian). The findings from our logistic regression analysis demonstrated no statistically significant differences in the baseline severity of the symptoms or the effects of TCHM administration when considering the predictors (Table 3).

### TCHM formulas and single herbs used for the management of COVID-19 sequalae in this study

Information regarding TCHM formulas and individual herbs was retrieved from the electronic medical records and categorized into three groups: respiratory, fatigue, and emotional distress symptoms. The most used formulas and single herbs are listed in Table 4 25-28.

## Discussion

In this hospital-based retrospective cohort study, we analyzed the clinical effects of TCHM in 47 patients with COVID-19 sequelae. The results revealed that personalized TCHM treatment regimen significantly alleviated fatigue and respiratory and emotional distress symptoms after 14 and 28 days. We suggest that TCHM administration should be considered an effective intervention for treating patients with COVID-19 sequelae.

Previous studies reported that TCHM treatment improved clinical symptoms, lung functions, and hematological parameters in patients with COVID-19 sequelae 11, 12. Li *et al.* demonstrated that TCHM administration resulted in notable alleviation of lung inflammation, respiratory, and fatigue symptoms, which were observed at the 84-day follow-up after hospital discharge in COVID-19 convalescent patients 29. Bu-Fei-Huo-Xue capsule treatments for three months were reported to attenuate fatigue symptoms and improve exercise tolerance with significant improvements in the 6-min walk distance and Fatigue Assessment Inventory scores in patients recovering from COVID-19 30. Our results demonstrated that TCHM treatment significantly improved post-COVID respiratory, fatigue, and emotional distress symptoms after treatment for 14 and 28 days. Furthermore, our results revealed significant improvements in both the physical and psychological fatigue symptoms after 14 and 28 days of TCHM treatment. Based on these results, TCHM administration demonstrated notable clinical effects over a relatively short intervention period.

The pathogenesis of COVID-19 sequelae is thought to involve multiple hypothesized mechanisms such as immune dysregulation, microbiota disruption, autoimmunity, abnormal clotting and endothelial function, and dysfunctional neurological signaling 5. Jiang *et al.* suggested that TCHM may contribute to the management of COVID-19 sequelae by relieving the convalescent symptoms, reducing organ damage, improving the repairment of organ injury, eliminating inflammation, regulating immunity, and alleviating coagulation abnormalities 31. Mukherjee *et al.* proposed herbal medicines and their phytocomponents manage post-COVID complications by modulating the immunological and inflammatory states 32. Certain notable single herbs used in this present study target the potential mechanisms of COVID-19 sequelae. Rhodiolae Crenulatae Radix et Rhizoma (Hong-Jing-Tian) shows anti-hypoxia, anti-inflammation, neuroprotection, and anti-fatigue effects 33-35. Ginseng Radix et Rhizoma (Ren-Shen) and Astragali Radix (Huang-Qi) exerts immunomodulatory, and anti-fatigue effects 36, 37. Angelicae Dahuricae Radix (Bai-Zhi) exhibits anti-allergic inflammation effects 38. Fermented Atractylodis Macrocephalae Rhizoma (Bai-Zhu) increases the distribution of *Bacteriodetes* and *Lactobacillus* (which may decrease after COVID-19 infection) 39 in the gut, as well as modulate microbial structure and gut permeability 40. Zingiberis Rhizoma (Gan-Jiang) shows anti-fatigue effects by exciting the adrenal cortex system 41 and regulates the abundances and interactions in the gut microbiota 42. Salviae Miltiorrhizae Radix et Rhizoma (Dan-Shen) protects endothelial dysfunction against mitochondrial oxidative stress, and exhibits cardiovascular and cerebrovascular protective actions 43. Acori Tatarinowii Rhizoma (Shi-Chang-Pu) exerts regulatory effects on the neurotransmitter levels, antioxidant stress responses, oxygen free radical scavenging, downregulation of inflammatory mediators, inhibition of neuronal apoptosis, and modulation of neuroglial cells and blood-brain barrier permeability 44. Polygalae Radix (Yuan-Zhi) demonstrates neuroprotective effects, including antioxidant, anti-inflammatory, neurogenesis, regeneration, differentiation, and neuronal plasticity enhancement 45.

Approximately 37% of the patients with COVID-19 sequelae had fatigue symptoms, including rapid physical exhaustion and chronic fatigue 4. Previous studies reported high degrees of similarities with the clinical manifestations of Long COVID and myalgic encephalomyelitis/chronic fatigue syndrome (ME/CFS) 46, 47, which is a multisystem neuroimmune illness characterized by a significant reduction in the ability to engage in pre-illness activities, profound fatigue, post-exertional malaise, cognitive impairment, and orthostatic intolerance 48. TCM provides strategies for treating patients with CFS in ancient texts and recent evidences 49, 50. A previous meta-analysis reported that application of TCHM on CFS results in significant improvements on the Fatigue Scale scores, Fatigue Assessment Instrument scores, Self-Rating Scale of mental state scores, Self-Rating Anxiety Scale, Depression Scale, and clinical symptom scores without serious adverse events.^51^ The underlying mechanisms of TCHM on CFS involves targeting regulating immune dysfunction, restoring the hypothalamic-pituitary-adrenal axis activity, and exerting antioxidative effects 49. Our results demonstrated that individual TCHM treatment regimen significantly improved post-COVID physical and psychological fatigue after treatment for 14 and 28 days. Although the relatively short period of fatigue symptoms in our study did not meet the criteria for CFS, the TCHM administration showed great clinical potential for treating CFS and post-COVID fatigue.

According to the World Health Organization definition, “Post COVID-19 Condition” is diagnosed at least three months after the initial illness, ruling out normal recovery time. Symptoms must last for two months to be considered as post COVID-19 condition. The symptoms can persist from the initial illness or develop after recovery, and a medical diagnosis is required to exclude similar conditions 52. However, most patients with COVID-19 sequelae in Taiwan visit the OPD or clinic less than three months after the initial illness. To alleviate the patients' symptoms promptly, implementing the strict definition of Post COVID-19 Condition in real-world settings can be challenging. In our study, the median duration from Dx to D0 was 37 days (IQR:19-73). The duration varied from 8 to 231 days. We performed logistic regression analysis using the duration from Dx to D0 (> 90 days vs. < 90 days) as predictors. No statistically significant difference was observed in the severity of the baseline symptoms or the effects of TCHM administration (CAT, CFQ-11, and BSRS-5 changes from D0 to D28) when considering this predictor. Moreover, no statistically significant differences were observed in the baseline severity of the symptoms or the effects of TCHM administration when other predictors were considered, including sex (female vs. male), age (≥ 60 years vs. < 60 years), and dietary preference (non-vegetarian vs. vegetarian). In summary, the examined factors did not have a significant impact on the baseline severity of the symptoms in the study cohort. TCHM administration showed consistent clinical effects regardless of the duration since initial illness, sex, age, or dietary preference (nonvegetarian vs. vegetarian). These results indicated that TCHM administration effectively exhibit beneficial clinical effects in diverse general populations with COVID-19 sequelae.

Patients with COVID-19 sequelae were observed to show "incomplete and delayed recovery" status. In TCM theory, this phenomenon was related to the interruption of self-healing ability after illness, which is important in the convalescence process to achieve complete recovery. We used the TCHM main formulas to stimulate the patients' self-healing ability and restore the impaired systemic balance primarily based on the Shang-Han-Lun framework 26. Single herbs are used to reinforce the effects of the TCHM main formulas and alleviate specific symptoms according to the TCM framework and modern mechanical research.

### Limitations

This study had several limitations. First, the number of patients in the study cohort was relatively small. Studies with a larger number of participants and more rigorous study designs are needed. Second, the specific mechanisms by which TCHM treats patients with COVID-19 sequelae remain unclear. Third, we retrospectively analyzed patients treated for 14 and 28 days. Studies with longer follow-up periods are warranted to evaluate the consistency of TCHM treatment effects and determine the recurrence of COVID-19 sequelae.

## Conclusions

Our study suggested that personalized TCHM treatment notably reduced respiratory, fatigue, and emotional distress symptoms after 14- and 28-days of treatment in patients with COVID-19 sequelae. The TCM strategies and treatment frameworks employed in this study offer valuable insights into the treatment of COVID-19 sequelae. Based on our findings, we propose that TCHM should be regarded as a promising and effective intervention for patients with COVID-19 sequelae.

## Figures and Tables

**Table 1:** Demographic and clinical characteristics

Characteristics	
**Demographics**	
Total, N	47
Age, years, median (IQR)	62 (49-70)
Duration of Dx to D0, days, median (IQR)	37 (19-73)
Duration of Dx to D0 > 90 / ≤ 90 days, n (%)	7 (14.9) / 40 (85.1)
Female sex, n (%) / Male sex, n (%)	32 (68.1) / 15 (31.9)
Age ≥ 60, n (%) / age < 60, n (%)	25 (53.2) / 22 (46.8)
Non-vegetarian, n (%) / vegetarian, n (%)	36 (76.6) / 11 (23.4)
**Clinical parameters, median (IQR)**	
COPD Assessment Tool, CAT, median (IQR)	14 (11-18)
The Chalder Fatigue Scale, CFQ-11, median (IQR)	17 (14-22)
Brief Symptom Rating Scale, BSRS-5, median (IQR)	5 (3-10)
**Biochemical and hematological parameters, median (IQR)**	
WBC, 10^3^/µL	5.67 (4.89-6.74)
RBC, 10^6^/µL	4.39 (4.16-4.83)
HGB, g/dL	13.3 (12.5-14.1)
HCT, %	40.6 (38.6-42.6)
PLT, 10^3^/µL	245 (210-281)
Neutrophil, %	56.1 (51.2-62.8)
Lymphocyte, %	31.9 (27.4-37.6)
Monocyte, %	6.2 (5.3-7.2)
Eosinophil, %	3.1 (1.8-4.1)
Basophil, %	0.5 (0.4-0.7)
Neutrophil-to-lymphocyte ratio, NLR	1.75 (1.37-2.27)
ESR, mm/hr	7 (3-10)
hs-Troponin I, pg/mL	2.6 (2.3-3.2)
CK-MB, ng/mL	1.1 (0.7-1.7)
P, mg/dL	3.3 (3.0-3.6)
Ca, mmol/L	2.34 (2.29-2.40)
K, mmol/L	4 (3.7-4.2)
Na, mmol/L	141 (139-142)
Albumin, g/dL	4.4 (4.3-4.5)
BUN, mg/dL	15 (12-18)
Creatinine, mg/dL	0.77 (0.64-0.93)
eGFR	88.74 (76.47-108.4)
Alkaline phosphatase, U/L	58.5 (47.25-64.25)
S-GPT/ALT, U/L	19 (13-27)
S-GOT/AST, U/L	19 (16-23)
CK, U/L	75 (54-115)
LDH, U/L	153 (138-167)

N, number; IQR, interquartile range; COPD, chronic obstructive pulmonary disease; D0, day of the initial visit to the TCM OPD; Dx, COVID-19 diagnosis date; WBC, white blood cells; RBC, red blood cells; HGB, HCT, PLT, platelets; ESR, erythrocyte sedimentation rate; hs-Troponin I, high-sensitivity cardiac-troponin I; CK-MB, creatine kinase-myoglobin; P, phosphorus; Ca, calcium; K, potassium; Na, sodium; BUN, blood urea nitrogen, creatinine; eGFR, estimated glomerular filtration rate; S-GPT, Serum Glutamic Pyruvic Transaminase; ALT, alanine transaminase; S-GOT, Serum Glutamic Oxaloacetic Transaminase; AST, aspartate aminotransferase; LDH, lactate dehydrogenase

**Table 2:** McNemar's test results

P value	CAT	CFQ-11	BSRS-5
**D0 vs. D14**	< 0.01**	< 0.001***	0.1489
**D0 vs. D28**	< 0.001***	< 0.001***	0.3865
**D14 vs. D28**	0.8137	0.7518	0.7237

** P value < 0.01, *** P value < 0.001. CAT, Chronic obstructive pulmonary disease Assessment Tool; CFQ-11, Chalder Fatigue Questionnaire; BSRS, Brief Symptom Rating Scale; D0, day of the initial visit to the TCM OPD; D14, day of follow-up visits to the TCM OPD 14 days after D0; D28, day of follow-up visits to the TCM OPD 28 days after D0.

**Table 3:** Logistic regression results

Predictor	Variable	Odds ratio	95% CI	P value
**Dx to D0 > 90 days vs. ≤ 90 days**	CAT on D0	1.0170	0.8865 to 1.155	0.8024
	CFQ-11 on D0	1.1150	0.9175 to 1.393	0.2822
	BSRS-5 on D0	1.0450	0.8856 to 1.217	0.5811
	CAT change	1.0780	0.9430 to 1.251	0.2756
	CFQ-11 change	0.9907	0.8458 to 1.172	0.9079
	BSRS-5 change	1.1850	0.9125 to 1.637	0.2244
**Female vs. Male**	CAT on D0	1.0470	0.9456 to 1.173	0.3849
	CFQ-11 on D0	1.0910	0.9433 to 1.277	0.2412
	BSRS-5 on D0	1.0920	0.9583 to 1.276	0.1954
	CAT change	0.9773	0.8799 to 1.081	0.6555
	CFQ-11 change	1.0050	0.8870 to 1.136	0.9337
	BSRS-5 change	0.9184	0.7366 to 1.105	0.3811
**Age ≥ 60 vs. Age < 60**	CAT on D0	0.9865	0.8959 to 1.084	0.7755
	CFQ-11 on D0	0.9152	0.7879 to 1.050	0.2090
	BSRS-5 on D0	0.9078	0.7952 to 1.022	0.1107
	CAT change	0.9640	0.8722 to 1.059	0.4476
	CFQ-11 change	0.9425	0.8334 to 1.057	0.3145
	BSRS-5 change	0.9872	0.8268 to 1.171	0.8804
**Non-vegetarian vs. Vegetarian**	CAT on D0	0.9681	0.8669 to 1.082	0.5584
	CFQ-11 on D0	0.8433	0.6874 to 1.002	0.0535
	BSRS-5 on D0	0.9197	0.8034 to 1.050	0.2113
	CAT change	0.9529	0.8449 to 1.065	0.3983
	CFQ-11 change	1.0790	0.9436 to 1.240	0.2656
	BSRS-5 change	1.0550	0.8635 to 1.282	0.5796

CI, confidence interval; CAT, Chronic obstructive pulmonary disease Assessment Tool; CFQ-11, Chalder Fatigue Questionnaire; BSRS, Brief Symptom Rating Scale; D0, day of the initial visit to the TCM OPD; D14, day of follow-up visits to the TCM OPD 14 days after D0; D28, day of follow-up visits to the TCM OPD 28 days after D0.

**Table 4:** TCHM formulas and single herbs for COVID-19 sequalae

COVID-19 sequalae	Main formula (Pinyin name)	Single herb (Latin plant name [Pinyin name])
**Respiratory symptom**	Gui-Zhi-Jia-Hou-Po-Xing-Zi-Tang	Trichosanthis Fructus (Gua-Lou-Shi)
	Gui-Zhi-Er-Ma-Huang-Yi-Tang	Fritillariae Cirrhosae Bulbus (Chuan-Bei-Mu)
	Gui-Zhi-Ma-Huang-Ge-Ban-Tang	Armeniacae Semen Amarum (Bitter Xing-Ren)
	Gui-Zhi-Jia-Ge-Gen-Tang	Platycodonis Radix (Jie-Geng)
	Ge-Gen-Tang	Astragali Radix (Huang-Qi)
	Chai-Ge-Jie-Ji-Tang	Rhodiolae Crenulatae Radix et Rhizoma (Hong-Jing-Tian)
	Xiao-Qing-Long-Tang	Angelicae Dahuricae Radix (Bai-Zhi)
	Ling-Gan-Wu-Wei-Jiang-Xin-Xia-Ren-Tang	
	Xiao-Chai-Hu-Tang without Ren-Shen, Da-Zhao, Sheng-Jiang, and add Wu-Wei-Zi, Gan-Jiang	
**Fatigue symptom**	Chai-Hu-Gui-Zhi-Tang	Ginseng Radix et Rhizoma (Ren-Shen)
	Xiao-Chai-Hu-Tang	Astragali Radix (Huang-Qi)
	Chai-Hu-Gui-Zhi-Gan-Jiang-Tang	Atractylodis Macrocephalae Rhizoma (Bai-Zhu)
	Li-Zhong-Tang	Cinnamomi Ramulus (Gui-Zhi)
	Si-Ni-Tang	Zingiberis Rhizoma (Gan-Jiang)
	Si-Ni-Jia-Ren-Shen-Tang	Aconiti Lateralis Radix Praeparata (Fu-Zi)
	Gan-Jiang- Ren-Shen-Ban-Xia-Tang	
**Emotional distress symptom**	Tian-Wang-Bu-Xin-Dan	Salviae Miltiorrhizae Radix et Rhizoma (Dan-Shen)
	Jia-Wei-Xiao-Yao-San	Acori Tatarinowii Rhizoma (Shi-Chang-Pu)
	Chai-Hu-Shu-Gan-Tang	Polygalae Radix (Yuan-Zhi)
	Yi-Gan-San	Ziziphi Spinosae Semen (Suan-Zao-Ren)
	Suan-Zao-Ren-Tang	Polygoni Multiflori Caulis (Ye-Jiao-Teng)
	Da-Cheng-Qi-Tang	Platycladi Semen (Bo-Zi-Ren)
	Da-Chai-Hu-Tang	Valerianae Radix et Rhizoma (Xie-Cao)
	Chai-Hu-Jia-Long-Gu-Mu-Li-Tang	Poria (Fu-Ling)
	Chai-Hu-Gui-Zhi-Gan-Jiang-Tang	
	Si-Ni-Tang	
	Si-Ni-Jia-Ren-Shen-Tang	

TCHM, Traditional Chinese herbal medicine; COVID-19, coronavirus disease-2019

## Abbreviations

ALT: alanine transaminase
AST: aspartate aminotransferase
BSRS-5: Brief Symptom Rating Scale
BUN: blood urea nitrogen
CAT: Chronic Obstructive Pulmonary Disease Assessment Tool
CFQ-11: Chalder Fatigue Questionnaire
CK: creatine kinase
COVID-19: coronavirus disease 2019
eGFR: estimated glomerular filtration rate
ESR: erythrocyte sedimentation rate
hs-Troponin I: high-sensitivity cardiac-troponin I
ICD-10: International Classification of Diseases, Tenth Revision
OPD: outpatient department
TCHM: traditional Chinese herbal medicine
TCM: Traditional Chinese Medicine
SARS-CoV-2: severe acute respiratory syndrome coronavirus 2
WBC: white blood cell count
